# Designed to Clash? Reflecting on the Practical, Personal, and Structural Challenges of Collaborative Research in Psychiatry

**DOI:** 10.3389/fpsyt.2021.701312

**Published:** 2021-07-07

**Authors:** Timo Beeker, Rosa Kato Glück, Jenny Ziegenhagen, Lena Göppert, Patrick Jänchen, Helene Krispin, Julian Schwarz, Sebastian von Peter

**Affiliations:** ^1^Department of Psychiatry and Psychotherapy, Brandenburg Medical School Theodor Fontane, Immanuel Klinik Rüdersdorf, Rüdersdorf, Germany; ^2^ExPEERienced – Experience With Mental Health Crises – Registered Non-profit Organization, Berlin, Germany

**Keywords:** collaborative research, psychiatry, user-involvement, participatory research, mental health, power asymmetries, co-production, research ethics

## Abstract

**Background:** In the field of mental health research, collaborative and participatory approaches in which mental health service users actively contribute to academic knowledge production are gaining momentum. However, concrete examples in scientific literature that would detail how collaborative research projects are actually organized, and how they deal with the inherent challenges are rare. This paper provides an in-depth description of a three-year collaborative project that took place in the wider context of a mixed-method process evaluation of innovative models of psychiatric care in Germany.

**Methods:** The in-depth description we provide here draws on a vast body of notes and records that originated from numerous meetings and sessions. The research group continuously and systematically reflected on their collaboration itself using the interpretative method of “interactive interviewing,” which included that also the personal memories of the researchers were collectively re-discussed before and during the process of writing. Our concrete experiences as a group were then contextualized with and analyzed in the light of more general challenges that are central to collaborative research in general.

**Results:** Performing collaborative research requires unconventional thinking and improvisation in order to find creative solutions for practical problems and to overcome the structural obstacles inherent to the process of academic knowledge production. An atmosphere of mutual trust and respect within the group is crucial, and continuous self-reflection or supervision can be largely beneficial. Challenges mainly originate from the vast heterogeneity that characterizes the researchers, usually including large differences in economic, cultural, and social capital.

**Conclusion:** Collaborative research in the field of psychiatry is designed to bring together researchers with widely diverse backgrounds. Emerging conflicts are important parts of knowledge production but also exceptional opportunities to negotiate research ethics, and potential vehicles for personal growth and transformation. Success or failure of collaborative research largely depends on how divergences and conflicts are articulated, mediated, and reflected. This also holds true in the light of the power asymmetries within the research team and the structural power inherent to the engines of academic knowledge production.

## Introduction

Internationally, there is a growing emphasis in research policy on the need for health service users to actively contribute to academic knowledge production (1). In mental health research, collaborative and participatory approaches in which persons with experiential expertise are part of academic research teams are gaining ground (2–5). The specific perspectives, competences, and experiences of user researchers are increasingly recognized as valuable sources of knowledge (6). As benefits in terms of research outcomes and practices seem to increase with the level of involvement, there are claims that researchers with experiential expertise should have decision-making power in all stages of the research process (7, 8).

Collaborative research can be demanding for everyone involved. The different contributors usually diverge in economic, cultural, and social capital as well as in perspectives and positions, which can challenge the realization of these kinds of projects (9, 10). Facing these difficulties, the scarcity of literature on how to concretely realize collaborative and participatory research is striking (7). The few and highly valued empirical studies in this field mostly focus on methodological aspects of the collaboration, therefore lacking insights on how the people involved concretely worked together. This information is usually condensed to either the confines of the method section or relegated to the gray literature, something that may also happen due to the high barriers for academic publication (6, 11). Existing guidelines in this field contain valuable advice on how to organize involvement or co-production, referring to processes of recruitment, payment, and training, as well as to ethical concerns, issues of occupational health, and career development (4, 12, 13). By means of generalization, these studies usually provide for rather technical, abstract information, often lacking detailed examples that would illustrate the everyday proceedings of the people and groups involved.

In more recent examples, the ways in which collaboration works have been explicated in detail: Lambert and Carr (7) elaborate on various ethical and organizational challenges they encountered while collaborating in a project that investigated the experiences of women with physical and mental health needs. They show how this kind of research often develops a dynamic of its own, potentially resulting in the appropriation of both the research methods and agenda by the participants. Rose and Kalathil (5) refer to several such projects, providing for detailed insights into the multifaceted challenges encountered when engaging in collaborative partnerships, such as the entrapment within fixed roles and positions, and the authority that is inherent to various forms of knowledge. King and Gillard (14) discretely reveal their experiences with this form of collaboration, elaborating on the concrete distribution (or ascription) of roles and identities in the course of a community participatory project that evaluated a primary mental health service. However, a slowly growing number of rather personal, highly interesting accounts about the personal challenges encountered in collaborative research has emerged during the past years. These accounts stem from various disciplinary fields and illustrate well what it concretely means to collaborate, mainly from the perspective of the researchers involved who had experiential expertise (15–21).

This article aims at adding to these contributions (and certainly others that are not known to us) by providing an in-depth description of our collaborative-participatory work together. This work took place in the course of a mixed-method process evaluation conducted within the wider context of a multi-centered, prospective, controlled cohort study, evaluating innovative modes of psychiatric treatment in Germany (PsychCare). Collaborative-participatory in our context means the joint knowledge production by researchers with and without experiential expertise of the psychiatric care system, of crisis and disability and recovery from them. This paper will have a double focus: In the first section, we will detail how our collaboration was realized and what helped us establish a productive and respectful work setting while being confronted with many practical obstacles. In the second section, we will discuss how we dealt with some of the inherent challenges of collaborative research, which were omnipresent or re-occurred over the course of our project. Discussion and Concluding Remarks will elaborate on additional aspects of these challenges, including an outlook on the role of power and on the impacts of our collaboration within our group and beyond.

## Context and Approach

### Context of the Study

The overall goal of PsychCare consisted of the comparative evaluation of the efficacy and efficiency of psychiatric hospitals which had implemented new “Flexible and Integrative Treatment (FIT)” strategies. Up to today, acute and intensive psychiatric treatment in Germany is predominantly provided in hospitals (22). Since 2013, a new legislation (§64b Social Code Book V) encourages FIT64b-models. All FIT64b models are provided for by a Global Treatment Budget (GTB), which is an annual lump-sum covering all hospital settings (23).

Currently, 22 German psychiatric hospitals have introduced FIT64b-models (24). So far, the GTB has mostly been employed to establish various forms of psychiatric day care, which can work as alternatives to conventional inpatient treatment, or to intensify outpatient treatment, e.g., through home-treatment. Building on previous, rather descriptive research projects based on FIT64b models, which showed overall positive results (24–33). PsychCare started as a prospective, controlled cohort study in 2017. The study was reviewed and approved by the ethics committee of the Dresden University of Technology. PsychCare also included a participatory form of process evaluation, following the Medical Research Council guidelines to evaluate complex interventions (34). All participants and interviewees provided their written informed consent.

### Context of the Research Group

The following material originates from our collaborative research during the three-year participatory process evaluation in the larger context of PsychCare. Our task was to assess users' experience with the FIT64b models. Our team consisted of three researchers with experiential expertise [experiential expert(s) = EE] of the psychiatric care system, of crisis and disability and recovery from them. One of the EE also held an academic degree. Furthermore, five researchers without experiential expertise [conventional researcher(s) = CR] collaborated in the project. Among these there were two MD students, two paid researchers who worked as psychiatrists, and one ethnologist. The latter's research agenda only partly overlapped with our core tasks. Therefore, she only sporadically attended the group meetings. Further expertise among the team members were degrees in anthropology and philosophy on the part of the CR. On the part of the EE, there existed work experience in a crisis resolution team, as a social worker, in handcraft and design, and a degree in social pedagogy.

The term collaboration, as we use it, neither intends to fully comply with the strict criteria of co-production (35) nor does it imply a systematic form of cooperation with actors in the research field in the sense of participatory research (6, 36). In the context of methodologically rather strict study designs, as was the case in our prospective, controlled study, collaboration designates an epistemic partnership that engages in common, systematic, and reflective efforts to expand forms of knowledge and practices on the part of the researchers involved. The various steps in which we organized our collaboration are described below. Our aim was to develop so-called experiential program components and a research tool based on them in order to evaluate the experiences and the fulfillment of needs during psychiatric treatment from the users' perspective. The work schedule of the process evaluation is displayed in Table 1, its results are published elsewhere (37, 38).

**Table 1:** Schedule of collaborative process evaluation.

**Stage of research**	**Timespan**	**Main activities**
Phase 1: Preparation	July 2017–August 2018	Construction of guidelines of the semi-structured interviews: 1) Re-analysis of 15 transcripts from a precursor-study (26) using a grounded theory based approach 2) Analysis and re-coding in several tandems of CR and EE 3) Deductive analysis of the transcripts (axial coding) by the CR 4) Generation of additional codes that related to the personal experiences of the involved EE = > Development of 12 experience saturated components of good care from the perspective of psychiatric users, serving as the main framework for developing (a) the guidelines of our semi-structured interviews, (b) the codes to analyze the empirical material, (c) a user-generated standardized research tool
Phase 2: Data collection	August 2018–January 2020	Conduction of semi-structured interviews: 5) In 7 psychiatric hospitals each, with and without ‘FIT’-elements (intervention group) by CR-EE-tandems in most of the cases to increase trust and openness of the participants
Phase 3: Data evaluation, interpretation, and publication	July 2019–December 2020	Analysis and interpretation of the interviews: 6) Coding of all interviews by either EE or CR, using the grounded theory methodology 7) Contrasting systematic differences between FIT- and Non-FIT-hospitals in the personal experience of users using Qualitative Comparative Analysis methodology 8) Publication of the main results (37, 38)

### Our Approach

In addition to our evaluative work schedule, we systematically reflected on our collaboration from the beginning, using the interpretative method of “interactive interviewing (39–42).” In “interactive interviewing,” participants act both as researchers and research participants by mutually interviewing each other about specific topics and personal experiences. The narratives produced in these interviews are collected, collectively re-discussed and interwoven with systematic reflections. The aim of this method is to reach an in-depth understanding of another person's experience and her view on complex, personal and sometimes sensitive matters. This can be the starting point for a reflection which proceeds from the specific, personal aspects to the construction of more general, abstract concepts, and theories.

For a short period, we used research diaries to document our efforts but had to acknowledge that taking notes separately did not advance a shared understanding. Instead, we started an open and ongoing reflexive process, consisting of special meetings, online discussions, and supervisory sessions for this purpose. We deliberated various ways of representing the products of this exchange, also involving ideas about multi-media means of representation to improve accessibility, such as video streams, podcasts, or comic strips. We opted for this rather classical publication format in order to place our ideas and experiences at the disposal of an academic readership. The following passages are based on our interactive interviews, which were documented in a vast body of notes and records. Also, during the writing process, many personal memories were exchanged and re-discussed in several feedback-rounds.

For heuristic reasons, we subdivided our findings into two parts: In the first section, a detailed description of our collaborative research process will be given. There we focus on some of the measures and strategies that proved valuable to us in tackling many of the unforeseen obstacles and problems that occurred during our collaboration. In the second section, we will reflect on our collaboration on a more abstract level. We assume that many of the challenges we recurrently confronted are part of the inner logic of collaborative research and, thus, can be understood as *inherent to* or *typical* of it. This section also demonstrates that collaborative research consists of much more than technically organizing knowledge production, and why it can be quite demanding on both the involved individuals and the research group as a whole.

Methodologically speaking, section 1 remains in a rather descriptive paradigm, mirroring the personal experiences collected through interactive interviewing. In section 2, description will mainly serve as the starting point for reflection and analysis as part of the more abstract, systematic engagement with the interview content. However, the material in section 2, which undeniably emerges from our own experiences, demonstrates that even the fundamental questions of collaborative research are inextricably interwoven with the personalities of the researchers and the interactions among them.

### A Terminological Caveat

We are aware that our use of the binary terminology of “EE” and “CR” is problematic in several ways. Firstly, it may easily tend to homogenize differing perspectives and positions *within* the two subgroups. These differed significantly along the lines of their diverse disciplinary and personal backgrounds, in addition to universal differences, such as age or gender. Secondly, the categorization as EE or CR risks being reductive: Every member of our group's identity comprised multiple layers or roles, among which just one consisted of being EE or CR. Furthermore, attitudes, personality traits, or ideas not originating from any contact with psychiatry occasionally may have had substantial impact on our work. Thirdly, it remains questionable if the label “EE” truly is a positive, empowering self-description, or a (potentially repressive) attribution by psychiatric professionals and academic scholars. An attribution that, furthermore, may tend to reproduce the traditional “doctors-patients”-divide. Fourthly, reproducing the binary terminology of EE and CR risks stabilizing these categories in discourse. In the worst case, this stabilization could also advance a naturalization, meaning that what is essentially a socially constructed divide could be mistaken for emerging from underlying (natural or biological) differences between two different kinds of human beings (19). Despite this critique, we decided to use this binary terminology as a starting point for further complexification and hope that our experiences with collaborative research, as they are described and analyzed below, can speak for themselves and in many ways challenge the EE-CR-distinction.

## Section 1: Organizing Collaborative Research

Within this section, we will describe what helped us establish and maintain our collaboration over the course of more than three years. The strategies and measures detailed below may neither be unique nor innovative. However, they proved effective in our individual case and may also be helpful in comparable work-settings. In their totality, they contributed to creating an atmosphere of mutual trust and respect within our group which might be crucial for the functioning of participatory and collaborative research groups.

### Work Environment

In many conventional psychiatric research projects, the work environment might be of minor importance. For instance, a workspace will be situated in a hospital setting for practical reasons. In our case, finding a suitable space required some effort. A main criterion for it was accessibility. As none of the EE did own a car and one was limited in the use of public transportation because of panic attacks, meetings in peripheral locations were not feasible. At the beginning, the only available room, which was accessible to all, was a vacant doctor's practice. This temporary solution turned out to be less than ideal. Even though it provided for a special atmosphere due to its abandonment, it still was a medical surrounding, and, thus strangely contrasting to the critical stance of our group toward medical institutions and rather hindering to emotional group processes.

When we had to leave this improvised location for budgetary reasons, we started to meet in the living room of one of our CR for a couple of months. This turned out to fit better, as the privacy of this space helped establishing an open but protected environment for dialogue. Furthermore, the mixed character of this room (it was originally used both as a working space and as a living room) resonated well with the hybrid nature of our relationship, falling somehow in between both professional and private. After some time, we managed to find a one-room-flat for rent that we used from then on as our office and as the location of our further group meetings. This apartment was part of a residential building, thus also satisfying our desire for a mix of both private and professional surroundings.

### Rules, Rituals, and Routines

Group meetings from three to five hours in length took place between one and three times a month, depending on the phases and tasks of the project. Every meeting began with all participants giving a brief account of their current mindset, mood, or feelings, which also served to find out if anyone of us was struggling with problems that limited his or her performance, or ability to concentrate, or required special attention and support from the group. A time schedule was brought together, structured by one of us who volunteered as host of the meeting, while someone else assumed the task of writing a protocol. Volunteers for both roles rotated between or within working sessions among both EE and CR.

Regular breaks every 60–90 minutes, or whenever needed, were part of every session. To ensure the accordance of our tempo, e.g., during discussions, to the needs of all group members, a green, a yellow, and a red object (three crocheted toy cars) were placed on the table. These were used in the sense of a traffic light system to indicate levels of distress, states of affection or exhaustion. The yellow object signaled the need for slowing down the tempo, or for more careful language, the red one fulfilled a veto-function, indicating that someone was highly irritated or unable to co-operate anymore, so that an immediate break or exchange could take place. To close off our sessions, either another round of spontaneous feedback took place, or each member of the group engaged in paying at least one compliment to every other participant, emphasizing individual strengths.

The totality of these routines and rituals enabled a joint process of working on, thinking about, and discussion of the complicated and often sensitive matters that arose within our group. They led to a safer climate—as discussions could be interrupted—and enabled the inclusion of heterogenous voices, also in states of upheaval or personal weakness. At the same time, they engendered a sense of playfulness and invited us to employ humor, irony, and kindness, all of which gradually developed into rather steady characteristics of our collaboration.

### Supervision and Exchange

As mentioned above, apart from our main work, regular group meetings on the topic of our collaboration took place every 8–12 weeks. These were complemented by supervisory sessions every nine months that were also explicitly dedicated to the discussion of our team atmosphere. The supervisory sessions were led by one or two supervisors with experiential expertise and long-time experiences with collaborative working relationships. They usually started with a separate supervision for the different subgroups (CR and EE), followed by a second part in which both subgroups were merged.

Both the meetings dealing with our collaboration and the supervisory sessions created an occasion to reflect on our working process on a meta-level. On an abstract level, we discussed topics such as the use of non-discriminatory language, questions of privilege, asymmetries of power, or our political positions. On a rather concrete level, we tried to deal with any disturbances, tensions, conflicts, hurt feelings, or misunderstandings that had occurred during the work sessions. Furthermore, we used these occasions to exchange- our individual motives for engaging in collaborative work.

### Tandeming

From the beginning on, tandems were an essential part of the implementation of our “collaborative” approach. Tandems were either loosely or more tightly bound couples of both an EE and CR, who worked together on specific tasks in various phases of our research process. At the beginning, these tandems enabled us to get to know each other, providing an opportunity to exchange details about our biographies and worldviews. Over the course of the project, this could also involve conversations dealing with sensitive topics, such as criticism of, or personal experiences with, the mental health care system, as well as the motivation to (keep) working in it. In addition to these relational aspects, tandems were the structural backbone of the co-working process between EE and CR in the various phases of the project. They provided for a safer space to exchange or integrate differing perspectives, to negotiate language, or to deepen understandings. Moreover, they served as an important vehicle to achieve mutual understanding on (potentially) conflict-laden topics: When tandem results were communicated to the entire group, they already had undergone a process of collaborative compromise, which often facilitated establishing agreements on the level of the group as a whole.

### Traveling

The tandems also proved to be valuable as a resource for the interview phase of our project that typically entailed several hours of traveling to, as well as a two or three-day long stays at, the research sites. Usually, the working tandems traveled together, thus allowing for an at least basic knowledge about the others' strengths, weak spots and preferences, and for an idea of how to get along best.

At times, traveling required special adjustments to ensure everyone's participation. To deal with the above-mentioned limited ability of one of our EE to use public transport, we rented cars or, if this was not feasible, carefully arranged joint train rides, avoiding the rush hours. Some of us had sleeping problems in foreign environments, especially in hotels. When overnight stays were inevitable, we thus relied, when possible, on private accommodation at friends' or acquaintances' places. On some occasions, we also rented flats with enough space to accommodate all of us.

Nonetheless, these research expeditions clearly constituted challenging and sometimes even extreme experiences for the EE. Fully recovering from them usually took several days if not weeks. Some of the EE had not been traveling for a long time due to anxieties and the need for a stable environment. The EE also were aware that elevated stress levels and lack of sleep, which both were very likely to result from such an expedition, could provoke new episodes of mental distress. Leading interviews in psychiatric hospitals also often implied a feeling of moving on difficult grounds which could trigger adverse emotions and memories (e.g., the sound of the locking door after entering the psychiatric ward). All in all, traveling often entailed a confrontation with one's own fears and the struggle to overcome personal limitations for the sake of the project. For instance, one of the EE with a history of claustrophobia managed to walk through a 200 m long tunnel deep below water, which separated the interview site from the accommodation for the night while being distracted by a casual chat about dogs with the CR-part of the tandem.

In the context of our research expeditions, the unequal distribution of resources between EE and CR became painfully visible. Although it was clear that all expenses would finally be covered, they had to be paid in advance, which regularly exceeded the budget of our EE members. Adding to this, refunds at times took six to nine months, and required us to overcome various bureaucratic obstacles. All our efforts to arrange easier procedures or an in-advance refunding system failed for administrative reasons. Ultimately, the leader of our department stepped in, when necessary, fronting costs with his private money.

### Dealing With Ups and Downs

During our collaboration, we experienced various up- and downturns, especially on the part of our EE members. All of them were living under socially precarious conditions that sometimes led to personal crises. They were sometimes also struggling with a lack of concentration or blurred thinking, e.g., due to the side-effects of their medication. Despite of that, the EE managed to work on an equal or even higher performance level than the CR, which contradicts widespread prejudices. At different phases, it even proved difficult for the CR to keep up with the commitment and enthusiasm of the EE, who sometimes seemed to work quantitatively more, and more in depth than their CR colleagues.

Nevertheless, the EE quite regularly communicated, for instance during the starting ritual, that they were not feeling well and were unconfident whether they would be able to concentrate or contribute that day. In these cases, the other members usually signaled their sympathy or willingness to provide support, when needed. It was also a clear rule that “dropping out,” either by leaving the room or by not paying attention, was always legitimate. Most of the time, special arrangements were unnecessary, and the contributions of the EE members turned out not to be affected at all. In other cases, small adjustments, such as the possibility of participating in the discussion with eyes closed or while lying on the couch, were sufficient to enable further participation. Moreover, encouraged by the group's supportive climate and openness for unconventional behaviors, one of the EE several times participated while struggling with what would qualify as pre-psychotic symptoms in psychiatric nosology. Interestingly, the CR gradually began to report when they were not at ease or in a crisis-like situation as well. For both subgroups, participation in our sessions increasingly contributed to a stabilization when they were not well by cheering them up or distracting them in a positive way.

However, the amount of invisible emotional labor facing the EE during our collaboration should not be underestimated. Traveling implied peaks of stress and sometimes triggered flashbacks or adverse emotions, which had to be regulated during the expedition and processed afterwards. Intense work sessions, e.g., during transcript analysis, could be exhaustive and deplete energy needed for other aspects of life. Moreover, dealing with one's own doubts and worries constituted an ongoing task. Due to their biographical backgrounds, all EE occasionally felt insecure whether they would be able to maintain a commitment to a long-term project, especially in the beginning. As confidence increased over time, new pressure to keep functioning arose from growing identification with the project and their own expectations. Thus, finding the balance between preserving one's (mental) well-being and the urge to push one's own limits, partly stemming from the dynamics of the project itself, remained a recurring challenge to the EE.

## Section 2: Inherent Challenges In Collaborative Research

After having described in section 1 how we set up a collaborative research process, we will now discuss some of the challenges that we encountered, either permanently or recurrently, over the course of our project. All of them touch on fundamental aspects of collaboration and, therefore, may be considered *inherent* to or *typical* of collaborative research. Of course, they do not necessarily appear in *all* collaborative research or work settings. Many of them can also be found in research or work settings which do not include EE members. However, they are part of the inner logic of collaborative research, which may help to shed light on what is really at stake, and how collaborative research is deeply interrelated with wider political and ethical questions.

### Heterogeneity

As mentioned above, our team comprised a vast *heterogeneity* of personal and professional backgrounds. Experiences, positions, and perspectives did not only differ along the EE and CR-divide but also within the two subgroups. As consequence of this heterogeneity, our engagement in collaborative research was fueled by a wide spectrum of motives and goals. Likewise, the perspectives toward psychiatry as an institution varied widely, ranging from a willingness to reform the current system to the wish to abolish institutional psychiatry once and for all.

This heterogeneity may be even broader in other projects, given that all members of our group were, for instance, white, grew up in a rather middle-class environment, and shared at least some basic political points of view (43). The resulting multi-perspectivity may be a characteristic feature of collaborative research as such, making it a trans-disciplinary scientific endeavor with all the inherent virtues and vices of transdisciplinarity (7). On the upside, the array of different skills and perspectives can be used as a valuable resource (44). On the downside, heterogeneity may easily erupt into conflict and significantly complicate the definition of collective goals.

To cope with the multi-level heterogeneity in our group, it was helpful for us to realize that our individual motives and interests did only need to be *transparent but not identical* to productively work on our task and advance a shared understanding. Full consensus on how to relate to the current psychiatric system or on the nature of our project's outcome was not needed for us to collaborate. Instead, our shared interest of working in a collaborative research group and the willingness to improve institutional psychiatry in its current state provided a solid common ground for our collaboration. Consequently, some fundamental differences in research interests, motives, and political agenda subsisted throughout the whole project and became tangible again, when we discussed the various outputs of our research (e.g., what to publish where and with which strategic purpose).

### Language

In collaborative research, language can be both one of the most powerful tools to create common ground and one of the most difficult terrains to navigate (15, 45). For our group discussions, as well as for the interview guidelines, and the publication of research results, we had to find a terminology which was at the same time clear and not discriminatory. Since many of the expressions in question were part of the specific discourse of the field of psychiatry, they, or their connotations, referred to broader concepts of what psychiatry is or is not, how it functions, how it should function, what psychiatric treatment consists of, and how it should be. Thus, minor linguistic problems could kick-start major ethical or political debates, and wording usually necessitated agreement in a much more fundamental sense, which made decisions sometimes painfully difficult.

For instance, it was relatively easy to agree on refraining from the use of medical terms and images, such as “mental disorder” or “illness,” to describe a person experiencing distress. Compared to this, it was more difficult to find an adequate alternative for the term “treatment” to name processes of change or growth which (potentially) took place during service use, since the term “treatment” is firmly linked with the biomedical paradigm and as such implicates a high degree of passivity on the part of the person being “treated.” Both aspects seemed not applicable to how personal change takes or took place according to the experiences of our EE members.

### Conflicts

Collaborative research projects are in a certain sense “designed to clash” for epistemological reasons, since they bring together researchers with heterogenic backgrounds. In addition, one of the sub-groups (EE) had often had markedly bad experiences with the profession represented by the members of the other subgroup (psychiatrists) in the past. Real transformation is not likely to happen without tensions (4, 45). When largely different perspectives and opinions collide, new insights may be gained, and new ideas may be generated. On the downside, conflicts within collaborative teams are likely if not unavoidable.

The most intense conflict that arose over the course of our project surfaced in a situation in which we discussed inclusion criteria for interviews. One of the CR argued for mainly including “the most severe cases,” contrasting them with “elderly ladies who just have a panic attack every now and then.” This opposition inadvertently dismissed one of the EE's personal problematic as a minor problem. After an intense argument, the person in question left the room and the group decided to terminate the meeting. Arguments, apologies, and efforts to reconciliate were exchanged over the following days in private and were also channeled back to the group. An extraordinary team supervision was organized, which provided a sufficient catharsis to continue with our work. In retrospective, the affected EE emphasized that without the private contact, and their^*^ previous experience of their^*^ CR colleague as being a fundamentally benign person, they^*^ might have left the project at this point. Thus, only personal ties enabled reconciliation and the continuation of our project, stressing the importance of allowing for hybrid, both personal and professional, relationships in collaborative work (46).

### Power Asymmetries Within the Group

Intrinsically connected to the above debates and conflicts, there were issues of power and power asymmetries in society and psychiatry as a whole, and the question if and how they were reiterated within our research group or its academic surroundings. The engagement with power in this twofold sense and how it is experienced during research can also be considered as a characteristic challenge intrinsic to collaborative projects (4, 7, 21, 43).

Our group consisted of individuals with extremely different shares in economic, cultural, and social capital, translating into different if not oppositional positions within the social hierarchy (47). These forms of capital correlated strongly with material privilege (e.g., working with unlimited contract, owning an apartment), or disadvantage (e.g., being unemployed, living on social security), and typically followed the CR-EE-divide. Moreover, power had played a central role in the life of all team-members in a very specific way: Everyone had personal experiences with the power of psychiatry, either being subjected to it at some point, or being an active part of its exertion.

Accepting our inability to reverse how the power of psychiatry and society had previously impacted our lives in very different ways, our more modest ambition was to reflect power disbalances in our interactions, decisions, and scientific work as much as possible. This included attempts to create an “open communicative space [36, p. 9]” or “ideal speech situation (48)” free from coercive influences and providing for the respectful exchange of opinions and arguments. Although an entirely equal distribution of the shares in verbal contributions was not pursued, with the help of the rotation of the function of moderator and regular feedback rounds we usually obtained a discourse in which no one felt continuously disregarded. Decisions were made on the basis of consensus whenever possible, which proved surprisingly successful. When majority decisions were necessary, they were made in a way so that the minorities could accept them as well. As much as temporary power vectors still appeared during our communication, they typically did not follow the CR-EE divide and emerged much more from individual properties, such as verbal persuasiveness, temperament, or expertise of the topic under discussion.

However, preexisting hierarchies prevailed as potential risks of undermining communication and decision-making. Among the CR, the team leader was also the direct clinical supervisor of the junior psychiatrist. While hospital work often requires top-down instructions, it had to be avoided in the research setting, which sometimes resulted in role confusions. Furthermore, conflicts originating from clinical work occasionally overshadowed cooperation within our group and even had to be, at times, mediated by the EE. Another asymmetry concerned the medical students, whose position in a traditional clinical hierarchy would be beneath that of the two physicians. In addition, they were the only team members who did not receive any payment for their work, which, unfortunately, still complies with German standards for medical dissertations. Among the EE, a potential asymmetry emerged from the fact that one of them, who had previously worked in a crisis resolution team, had been in a therapeutic relation with another one of the EE up to only a few months prior to the beginning of our work. However, neither them nor the rest of the group had the impression that their collaboration was affected by this previous relation.

### Structural Forms of Power

In addition to the asymmetries within our group mentioned above, we were also confronted with rather exogenic, structural power imbalances, which already became tangible during the formation-phase of the team: The initial concept for the team was to install two *user researchers* with an academic degree as EE to create an equilibrium of formal qualification between the CR and EE subgroups. After difficult negotiations, the concept had to be modified, which was partly due to bureaucratic obstacles to the provision of an adequate salary for the EE. The user researchers in question decided not to accept the job and were replaced by three peer researchers. This position is commonly defined as researchers with lived experience, but without academic qualification, which was also reflected in their salaries. Although one of them held an academic degree, this person was, for budgetary reasons, nevertheless employed as a peer researcher—a fact on which we as a team had no influence. This example represents a widespread pro-academic bias, according to which formal qualification best guarantees for competence, while informal qualification, such as lived experience, is disregarded (49, 50). Since this bias is also engrained in the engines of academic research, we assume that being discriminated by, and having to struggle against, this kind of structural power are generalizable challenges intrinsic to collaborative research (12, 43, 45).

A second example for structural forms of power is the influence exercised by the overall PsychCare study on our sub-project. As mentioned, collaboration between CR and EE exclusively took place in our part of the study. For various reasons, only the leader of our group stayed in contact with the leaders of the other study parts. This led to an implicit, and often also explicit, hierarchy, since the leader was the only one who had direct access to the information that circulated within the overall consortium. Moreover, this very leader of our group also was the person with the largest research experience among us. Consequently, he was often the one who gave decisive advice on which parts of the generated knowledge were to be regarded as useful or ready for further processing. In doing so, however, he himself followed rather rigid academic rules and parameters, being in a subordinated position to the authority of scientific knowledge and academic knowledge production. For instance, being the leader of the research group, he had to secure future research funding by generating as much academic impact as possible.

## Discussion

The cooperation with EE in research projects still is rather uncommon in psychiatry, especially when research follows the biomedical paradigm. Working in a collaborative project, thus, can be seen as an occasion in which *unusual* work-relations are established. For instance, they are unusual because of the wide heterogeneity within the group, the very different experiences with psychiatry that underlie individual engagement, the large differences in socioeconomic status and privilege, and, interdependently, the potential collision of individual worldviews. Under these circumstances, the formation of a well-functioning team and the development of a sensitive, deeply respectful team-culture can be considered as an ongoing challenge but also as an opportunity that harbors huge potentials for personal transformation.

As shown in the first section, how to organize a research expedition or a group meeting in collaborative research may necessitate more cautious thinking than in more conventional projects. Instead of providing for a generalized “how-to-guide” for collaborative research, we tried to describe some of the experiences of our three-year long research journey. In line with Carr et al. [45, p. 1], who insist that “there is no single, universal model of co-production,” we reckon that every collaborative project will have to find its own ways, depending largely on the object of research, methodology, available resources, configuration of the group, properties of individual group members, and many more of the parameters that directly and indirectly determine its context. This precludes any form of manualizing or formalizing of collaborative approaches. In a similar vein, we intended to emphasize that performing collaborative research requires unconventional thinking, improvisation, and the willingness to find creative solutions in order to handle the structural and other kinds of obstacles that usually spontaneously occur over time. As an attempt to bridge the gap between such rather theoretical statements and collaborative research in practice, the first section of this article detailed several examples of the specific problems that emerged during our project and the tools that helped us to solve them. In this context, a special focus was on the perspectives of the EE. Adding to the rather scarce literature on this topic, we highlighted the amount and the different dimensions of emotional labor that even positive identification with a collaborative project may entail for EE, be it while traveling with public transportation or entering a locked psychiatric ward as a researcher (16, 45). In line with the widespread claim that *trust* is paramount for collaborative research (4, 21, 44, 45), we also provided some intimate insights into the situations through which relations of trust were built in our case and how those relations proved essential to maintaining collaboration when difficulties arose.

As demonstrated in the second section, the ways in which our collaboration itself became a research topic opened up the space for an enriched and deeper understanding of some inherent and probably generalizable challenges facing collaborative research. Substantial heterogeneity of the team members can be a mixed blessing, both being a motor for productive discourse and a source of conflict. To value heterogeneity as a resource, it is important that all researchers involved bring to the fore an attitude of fundamental openness to diverging perspectives, a willingness to engage in an ongoing dialogue, and a continual process of (self-)reflection (44). As Roper et al. [4, p. 2] coin it, collaboration can only succeed embedded in a “culture that embraces exploration and learning.” According to our experience, this culture may only thrive when it includes a certain readiness to allow interpersonal relationships of a hybrid nature located in between the professional and the personal.

Heterogeneity is also directly linked to a particular notion of collaboration: Since collaborative research occurs among persons with (often extremely) different shares in economic, cultural, and social capital, a high divergence of interests, competencies, and motivations is often at play as well. Collaborative projects, thus, are *designed to clash*, as tensions and frictions are likely to appear, but serve as important vehicles toward a shared understanding and personal growth. In parts of the existing literature, there is a tendency to perceive conflict as something that must be expected but is intrinsically undesirable and should be avoided if possible (4, 44). Far from advocating disrespectful or careless interactions in collaborative research, we would argue that it is important to acknowledge and value the opportunities of substantial epistemic gains that lie in the numerous tensions and frictions that may become manifest during the research process. Consequently, we would suggest that collaboration may be best understood as a joint epistemic work that inherently takes into account heterogenous interests, disciplinary contingencies, and forms of knowledge but does not aim at their synthesis and has a high tolerance for disagreement. Understood in such a way, the most important challenge of collaboration is to continuously reflect these underlying differences and asymmetries. Furthermore, although its epistemic benefits should be embraced, it is necessary to mediate conflicts among the participants, and to build up a research process based on *equality in rights* but without denying the existence of privileges, of disadvantages, or of mere difference (21, 45). Thus, we suggest conceiving of collaborative projects as *practical experiments* in both *research ethics* and *ethical research*. Hence, they are *designed to clash* also in the sense that they may—unsurprisingly often—be colliding with the structural forms of power that are engrained in the institutions and practices of academic knowledge production.

### Personal Growth

In the tradition of social constructivist approaches to science, it is a widespread position that the results of research (or: *the construction of a research object*) are inevitably shaped by the person of the researcher. In collaborative research, this relation may partially be turned upside-down: the research process and the collective, discursive construction of the research object (e.g., through permanent reflection and debate) here also *shape the researchers*, thereby opening up a path for personal transformation. To summarize in an—admittedly—simplified formula, one can say that this personal growth occurred in our project in *stabilizing* (e.g., in terms of self-confidence, well-being, social situation) the EE members of our team, while productively *labilizing* the professional habitus, attitudes, and previously unquestioned points of view among the CR. At first sight, this formula seems to resonate well with Roper et al.'s statement that “non-consumer partners (in our case: the CR) may need support to position themselves as learners and consumer partners (in our case: the EE) may need support to position themselves as leaders within co-production groups [4, p. 8].” At second sight, our experience rather suggests that *all of us* may have been learners and also, all team-members may have acted as leaders in some respect during our three-year collaboration.

For the two CR who also worked as psychiatrists, the ongoing reflection in the course of the research process prompted a learning process which led to a better understanding of their personal motives for working in the field of psychiatry and in a collaborative research team, respectively. Moreover, they experienced the reflections of power asymmetries as occasionally eye opening with regard to their own subtle privileges and the disadvantages faced by people with a history of psychiatric treatment. Apart from that, highly individual aspects emerged. One of the CR gained access to a new level of understanding of how feelings of shame and guilt, stemming from his family history, motivated his collaborative work. Another described how the collaboration had labilized his clinical routines, prompting, for instance, a process of making his feelings in clinical conversations more transparent. He also gradually started working in a less directive way, e.g., by rather asking for needs than suggesting solutions.

In similar ways, the EE experienced positive changes caused by our collaborative project, an effect of collaborative research that has also been described in the existing literature, where it is sometimes rubricated under the term “empowerment (7, 36, 44, 51).” In our case, the EE expressed that the project had led to the realization that their seemingly “private” knowledge constitutes a valuable resource for research and could be useful to many other users as well. This also meant that they were not just people who had experienced psychiatry but skilled experts or even professionals themselves. Both points constitute a gradual change of view, that can also be understood as a process of learning. In addition, working and, especially, traveling together often induced the EE to confront their own fears and to push their own limitations in a way which resulted in feeling more self-confident or even in regaining more freedom in everyday life. Furthermore, allowing the development of a trustful relation with psychiatrists was a surprisingly new experience for some of the EE. The close and ongoing exchange with the CR who worked as psychiatrists contributed to a clearer picture of the pressures and necessities in the background of the clinical setting, and of how little freedom of action psychiatrists sometimes possess within the overall structure of rules and routines which comprise psychiatry as a highly institutional and commercialized establishment.

### Reflections on Power, Power Asymmetries, and Potentials for Change

As described by other authors as well, the ongoing engagement with power might be a core feature of collaborative research (4, 7, 45, 52). Power is pervasive and omnipresent in collaborative research, making it impossible to ignore. As Lambert and Carr put it, “power and control are inherent in the research process and that it is all our responsibilities to manage it ethically [7, p. 7].” Accordingly, collaborative research may be a field in which many of the often-subcutaneous aspects of power in psychiatry and society as a whole *crystallize* and thus *become visible*. Hence, the omnipresence of power in collaborative research might *rather constitute an opportunity than a challenge*, opening up a laboratory for observing social power at play and for experimenting with which aspects of it can be mediated or deconstructed and which cannot. In this sense, we attempted, and often managed to, reduce the influence of power asymmetries on our work by continuously reflecting and consciously trying to counteract them. Thereby the whole group benefited largely from the EE' sensitivity for power, safeguarding that temporary power vectors did not evolve into permanent domination.

At the same time, and somewhat paradoxically, we realized that the mere intent to collaborate on the content-level may lead to a tendency to gloss over differences in opinion, dissent, and interpersonal tensions. All of these need time, space and occasions in which they can be articulated and—as far as possible—mediated. Collaborative projects thus might quite often have to navigate the narrow course between Scylla and Charybdis: On the one hand, if subtle discord and disagreement are denied or “glossed over” and, thus, remain silent in the subconsciousness of the research process, important aspects about collaboration itself may be overlooked. Slight feelings of discomfort can be hints for the perseverance of power asymmetries and discrimination, which may potentially erupt into open conflict sooner or later. On the other hand, as collaborative research projects are usually devised to fulfill a specific task in a bigger machinery of knowledge production (in our case we conducted a sub-project tasked with user evaluation which was subordinated to a larger study), a focus on content-related work is to a certain degree inevitable. Moreover, making content-related progress usually boosts a positive group identity. Hence, dedicating too much of the limited timeframe and financial budget to group-dynamics and reflections might lead to stagnation of the research process and to personal frustrations. Thus, this can culminate in running the risk of a failure of the project as a whole, which, from a strategic point of view, will decrease the likelihood to find funding for more collaborative projects in the long run.

## Concluding Remarks

Despite all efforts to mitigate power asymmetries and their effects, it seems unrealistic to us to expect that collaborative research could take place in a self-created power-vacuum. This is especially true when power is understood from a Foucauldian perspective not as direct coercion but as pervasive and subtle, embodied in discourse and individual identities (53, 54). Our overall experience was that our research often felt like it was taking place in an artificial space in which the real-world differences between us in terms of power or possessions *felt less real* and less threatening, and where we could at least temporarily act *as though* we were all equal in every relevant aspect. In this sense, collaborative work might have provided us access to what has been coined as *heterotopia* by French philosopher Michel Foucault, a socio-cultural “other-space (55),” which is an approximation toward an ideal or maybe even utopia. A heterotopia in this sense is a world in a world. It is at the same time absolutely real for the persons who experience it while they are experiencing it and unreal in comparison to the order of the society surrounding it. Assuming that collaborative projects can be conceived of as taking place in such a heterotopia, one fundamental question would be if (and how?) the quasi-utopian experiences made in this other-space can be transferred into the real world and translated into real social progress.

In our case, the answer would be deeply ambivalent: Our attempts to reverse the power asymmetries between CR and EE which are engrained in the structures of the overall study, and in academic administration as such, were clearly of limited success. Also, the economic inequality between CR and EE increased steadily over the three years, as academic payment structures determined that the EE were paid significantly less than the CR. On the other hand, our results will most likely contribute to bringing about a reform of mental health care toward approaches which are more emancipatory in some aspects, firmer grounded in human rights, and which may even serve to counteract the progressing psychiatrization of society (56, 57). Furthermore, although the material gap between CR and EE could not be closed, our project opened up what could be called a new career path for all three EE: Even prior to the official termination of PsychCare, all of them had signed contracts in successive projects. Due to an involvement with more work hours and a higher salary, one of the EE will even manage to escape from the often intrusive German social security system.

## Author Contributions

TB and SP were responsible for devising the article. TB wrote the initial draft and coordinated the other authors' contributions. All authors contributed to interactive interviewing, literature search, interpretation of literature, helped draft the final version of the manuscript and revised the article critically for important content. All authors approve the final version to be published and agree to be accountable for all aspects of the work, its accuracy and integrity.

## Conflict of Interest

The authors declare that the research was conducted in the absence of any commercial or financial relationships that could be construed as a potential conflict of interest.
